# The Value of Phenotypes in Knee Osteoarthritis Research

**DOI:** 10.2174/1874325001812010105

**Published:** 2018-03-16

**Authors:** Fred R T Nelson

**Affiliations:** Department of Orthopaedics, Henry Ford Hospital, 2799 West Grand Blvd. Detroit Michigan 48202, USA

**Keywords:** Osteoarthritis, Physical features, Phenotype, Imaging, Progression, Knee, Phenotype, Classification Phenotype in knee osteoarthritis

## Abstract

**Background:**

Over the past decade, phenotypes have been used to help categorize knee osteoarthritis patients relative to being subject to disease, disease progression, and treatment response. A review of potential phenotype selection is now appropriate. The appeal of using phenotypes is that they most rely on simple physical examination, clinically routine imaging, and demographics. The purpose of this review is to describe the panoply of phenotypes that can be potentially used in osteoarthritis research.

**Methods:**

A search of PubMed was used singularly to review the literature on knee osteoarthritis phenotypes.

**Results:**

Four phenotype assembly groups were based on physical features and noninvasive imaging. Demographics included metabolic syndrome (dyslipidemia, hypertension, obesity, and diabetes). Mechanical characteristics included joint morphology, alignment, the effect of injury, and past and present history. Associated musculoskeletal disorder characteristics included multiple joint involvement, spine disorders, neuromuscular diseases, and osteoporosis. With the knee as an organ, tissue characteristics were used to focus on synovium, meniscus, articular cartilage, patella fat pad, bone sclerosis, bone cysts, and location of pain.

**Discussion:**

Many of these phenotype clusters require further validation studies. There is special emphasis on knee osteoarthritis phenotypes due to its predominance in osteoarthritic disorders and the variety of tissues in that joint. More research will be required to determine the most productive phenotypes for future studies.

**Conclusion:**

The selection and assignment of phenotypes will take on an increasing role in osteoarthritis research in the future.

## 1 INTRODUCTION

The Merriam-Webster dictionary definition of phenotype is “the observable properties of an organism, which are produced by the interaction of the genotype and the environment.” In osteoarthritis (OA) research, the term has been used increasingly to define physical, biochemical, and genetic characteristics along with physical events occurring in both past and present. This review relied on the expanded use of the term phenotype as it is applied to OA research. There are organizations that have suggested key knee osteoarthritis (KOA) phenotypes. In a recent review, pain sensitization, psychological distress, radiographic severity, body mass index (BMI), muscle strength, inflammation, and comorbidities were associated with clinically distinct phenotypes [1]. These along with gender, obesity and other metabolic abnormalities, and pattern of cartilage damage were evaluated by a literature review to determine any validation of these phenotypes [1]. This review highlights a number of additional OA phenotypes that could be used depending on the type of progression or the nature of a therapy being investigated and the population under study. Appropriate selection of phenotype could lead to a better understanding of the role of a specific phenotype or phenotype cluster relative to disease progression as well as treatment outcome.

The term personalized medicine is not the same as concierge medicine. Concierge medicine delivers care separate from concerns about insurance and reimbursement. The term personalized medicine is treatment based on specific biomarkers or characteristics that might define the peculiar nature of the recipient of care. This increases the chance of not missing an effective therapy in a heterogeneous population [2]. The Subpopulation Treatment Effect Pattern Plot (STEPP) approach was developed to allow researchers to investigate the heterogeneity of treatment effects on survival outcomes across values of a (continuously measured) covariate, such as a biomarker measurement [2]. In the case of KAO, there are several predetermined approaches to a more appropriate selection of therapy for any given individual. Hence, the selection of the most appropriate phenotype for any given OA study aides both the clinician and the scientist.

The bulk of therapies for OA are directed at pain relief, and although many are evaluated for disease modification, so far there has been no long-term demonstration of impact on disease progression. However, weight loss has been shown to slow the rate of joint deterioration over a period of 4 years [3]. Many studies show delay to total joint replacement but do not show imaging or other evidence of delayed disease progression. Given the myriad pathways to articular cartilage degradation, the exploration of specific genetic targets has expanded exponentially. With the completion of the human genome, improved proteomics, and more efficient and less costly mRNA and DNA analyses, including microarrays, many biomarkers for OA progression have been explored. There is no specific single gene product that has been found to affect a large majority of patients with OA. Using microarray technology, investigators have turned to the discovery of gene clusters that may identify more rapid OA progression. A problem for any systemic intervention is finding an impact on the specific disease without an impact on the rest of the body. A corollary is finding a gene or gene cluster that targets only 1 tissue. The Osteoarthritis Research Society International (OARSI) has developed guidelines relative to the use of soluble biomarkers in OA research. Within that guideline was the notation that identification of the biomarker may be as soluble or ‘wet’ (biochemical analyte, genomic, *etc*.) or as non-soluble or ‘dry’ (imaging, a physical examination finding, scales, *etc*.) [4]. The 5 “dry” clinical phenotypes recommended by OARSI for use in KOA studies are listed below.

1. Previously uninjured men and women, aged 25 to 35 years old, with acute anterior cruciate ligament injury (< 4 weeks old) with or without primary arthroscopic ligament reconstruction or meniscectomy. The status of other joints is normal.
2. Postmenopausal women with symptomatic and disabling (> 6 months) bilateral multi-joint erosive hand OA as detected by magnetic resonance imaging (MRI); other joints are asymptomatic.
3. Individuals aged 40 years old or older with new-onset unilateral knee pain (< 6 months) without knee trauma, but with normal knee radiograph. Knee MRI shows meniscal pathology (extrusion/degenerative tear), subchondral bone marrow lesion, effusion and/or minor tibiofemoral cartilage defect(s).
4. Symptomatic and radiographic KOA.
5. Symptomatic and radiographic hip OA.

Using a phenotypic and biomarker approach could help clarify controversial areas of KOA treatment. Several popular therapies for OA pain, viscosupplementation, and glucosamine, have come under scrutiny and have been declared “not recommended” by large medical organizations [5]. However, this does not take into account heterogenicity and individual population selections [6]. In addition, there is a call to give attention to different phenotypes of KOA since they may respond differently to each treatment [7]. By example, this is discussed on the subject of viscosupplementation in a younger military population [8]. Although pain does not correlate to imaging studies, the opportunity to intervene in the early phases of the disease is highlighted by the spectrum of different tissues that are initially involved in the degenerative process. The presence of meniscal change, bone marrow edema, synovitis, and infrapatellar fat pad (Hoffa’s fat pad) synovitis have been found to be predictive of radiographic change within 4 years [9]. The targeting of tissues other than articular cartilage may be an important consideration in the development of therapies for treating KOA. This is particularly important in those cases where radiographs appear normal.

Over the decades the term phenotype has changed in its connotations. When applied to inherited disorders, the term phenotype was applied to the physical appearance or clinical presentation of a person with an inherited disorder. The term penetrance was applied to the variation as to the severity of phenotype relative to normal. Over time it has been found that penetrance was often a reflection of different mutations for the same gene or a mutation in more than 1 specific gene that could contribute to a similar phenotype. The expansion of the term phenotype to research applications was highlighted in an editorial on the spectrum of phenotypes for investigation of OA [10]. This included age, estrogen status, genetic factors, components of metabolic syndrome, specific tissues affected, serum biomarkers, the shape of joint, joints affected, and the stage of disease [10]. A separate recommendation by OARSI was based on an Amsterdam cohort study listing 5 phenotypes more likely to be productive in research [11]. These were “minimal joint disease phenotype,” “strong muscle phenotype,” “non-obese weak muscle phenotype,” “obese and weak muscle phenotype,” and “depressive phenotype.” In some quarters the term phenotype has expanded to overall health, disease history, behavior, and general disposition. With the advent of electronic records, retrieving some of these details is becoming more practical. However, in research design, it is very easy to overlook easily observed and recorded features that may prove important in finding responders versus non-responders to various therapies. Much of this information can be obtained noninvasively, allowing for better determination of individuals with a favorable response to a treatment that is ineffective for others.

## 2 METHODS

A search of PubMed alone was used to find all articles relative to KOA and phenotypes. The search included “knee AND phenotype” and “osteoarthritis AND phenotype”. Once these were compiled, 4 groups of characteristics were created. The focus of this review is on the knee. Because the mechanical effects of deformity for the hip and knee have an overlapping impact, both joints will be considered in the section on mechanics Table (**1**).

## 3 RESULT

Having determined the range of suggestions of specific phenotypes in KOA, this review was grouped into 4 sections:

1. Demographic characteristics including components of metabolic syndrome.
2. Mechanical characteristics including joint morphology, alignment, the effect of injury, and history of present and past activity on affected joint.
3. Associated musculoskeletal characteristics including multiple joint involvement, spine disorders, neuromuscular diseases, and osteoporosis.
4. Joint tissue characteristics including cartilage, meniscus, synovium, bone sclerosis, bone cysts, and osteophytes. Included was the location of pain such as the joint line, focal synovial, bone, and global could indicate the involved tissue(s).

## 4 DEMOGRAPHIC CHARACTERISTICS

*Height and weight.* Demographics that may influence OA development and progression are age, sex, race, height, and weight. The latter two are incorporated into a crude measure of proportional body fat, BMI. BMI is the key component of metabolic syndrome, which also includes diabetes, dyslipidemia, and hypertension. Recently the addition of KOA and hand OA to this list has been recognized [12]. An elevated BMI is associated with OA progression. The inflammatory effects of fat are thought to be an important factor [13]. There is also an association of KOA with leptin, which is elevated in some obese patients [14, 15]. This is a reflection of the relevance of obesity in the OARSI phenotype recommendations.

*Sex.* Many studies on intervention for knee pain show that women are more prevalent than men. In a meta-analysis of 46 out of 6554 screened papers, it was determined that individuals aged 50 and over and who were female gender had a pooled odds ratio of 1.68 (95% CI 1.37-2.07). For contrast, the pooled odds ratio for obesity was 2.66 (95% CI 2.15-3.28), making obesity a more relevant factor [16].

*Ethnicity.* The role of ethnicity in OA has been studied by the National Health and Nutrition Examination Survey I, which suggested that African-American women are more likely to develop KOA than men and white persons [17]. However, in the Johnston County Osteoarthritis Project, there was no evidence of ethnic differences [17, 18]. The results are quite the opposite in hip OA, where National Health and Nutrition Examination Survey I did not reveal differences [19] whereas the Johnston County Osteoarthritis Project did [20]. It probably depends on lifestyle, socioeconomic factors, BMI, and even genetic factors within different geographic locations [21].

*Age.* Age is known to be associated with the development of OA. The long half-life of type II collagen, the major collagen of cartilage, is decades [22]. However, during this time there is cleavage of collagen without complete degradation [23]. The combination of the loss of tensile properties, passive glycation, and accumulated collagen cleavage lowers the functional capacity to accept forces that were tolerable decades before. In cartilage, type II collagen has high tensile properties. Peaking at age 30, in many individuals, the tensile properties then begin to diminish [24]. It has been found that increase advanced glycation end products in collagen contribute to a loss of tensile property [25]. Overall, the cleavage of collagen results in loss of tensile properties [26]. In a review on the effect of age on cartilage matrix, cells and the development of OA, it is clear that there are fewer cells and that damaged telomeres result in a senescent secretory phenotype [27]. This degradative phenotype has increased production of cytokines including interleukin-6 and -1 and matrix metalloproteinases. Advanced glycation end-products make for a more brittle type II collagen leading to brittleness and fatigue and failure [28]. Similarly, there is fragmentation and eventual loss of aggrecan. The combination of increased reactive oxygen species is directly related to intracellular proteins and extracellular matrix.

## 5 MECHANICAL CHARACTERISTICS

*Knee noninjury.* Varus and valgus deformity of the knee has long been associated with medial versus lateral compartment KOA. It has also been noted that increased adductor moment is also associated with KOA. Adductor moment is the relative medial directed distal tibial motion following heel strike. This results in increased force on the medial compartment. The moment force can be calculated integrating the total force over time. A component of adductor moment force is the maximum impulse, which is the peak force that occurs during stance. Cumulative adductor load is the product of the total adductor moment force multiplied by the number of steps. Cumulative and peak adductor load was higher in an OA group versus a non-OA group and cumulative load was the best predictor [29]. In a Japanese population, the combination of varus thrust and degree of varus deformity has the greatest correlation to OA [30]. The term impulse has also been applied to a brief acceleration of acceleration that may be high in individuals who are termed impulse loaders. This occurs right after heal strike and as a stand-alone measure may be related to the development of KOA [31]. Knee flexion-extension, abduction-adduction, external-internal rotation, lateral-medial translation, anterior-posterior translation, and superior-inferior translation are involved in activities of daily living and thereby influence the resultant joint reaction force. So far, these have not been placed in a discussion of phenotypes.

Similar to the hip, there is a variety of knee shapes that may be related to the development of OA. Shape variations can result in areas of force concentration. One approach to the impact of shape is the use of axial slices obtained by using MRI slice reconstruction and comparing shape in OA and non-OA individuals [32]. MRI slices in patients who had a joint replacement were compared to the opposite knee if asymptomatic at the end of the study and to control subjects with no knee pain. Calculated variations in shapes correlated with progression to TKA [33].

*Knee injury.* Direct injury to articular cartilage or changes in the mechanical integrity of the joint following meniscal or ligament injury can have both an immediate and long-term effect [34]. These injuries often result in hemarthrosis, chondrocyte death and bruising of the underlying bone. There is a release of inflammatory mediators in the acute post-injury period. These responses often improve over the next 3 to 6 months, but the long-term residuals become apparent later. Repair of meniscal and ligament damage may improve function but does not prevent the long-term effects of the initial injury to the articular cartilage. The long-term response is influenced by the initial degree of articular cartilage damage, the genic profile of the individual, joint stability, and metabolic factors such as obesity. Detection of an injury phenotype may be disguised by recovery from an initial impact wherein the person has long since forgotten the injury. This is particularly true of contact sports participants.

The occupational load history of the joint is significant. By review of the literature, it is apparent that activates involving lifting while squatting are associated with KOA [35]. The incidence of KOA in farmers, construction workers and forestry work has increased. This is also true for hips, particularly in occupations requiring many stairs or ladders. The number of years in these activities has an accumulative effect. There are numerous avocational bent knee activities such as household chores and avocational sports wherein the forces are identical. It has been shown that in the face of knee symptoms, reducing the force or frequency of some activities will help resolve symptoms [36]. As part of the mechanical phenotype, obtaining a retrospective analysis of cumulative loads may not be accurate. A more precise evaluation of the length of time a person has been in specific vocations may shed light on the higher rate of KOA and hip OA in those populations.

*Hip morphology.* The term idiopathic OA of the hip began to be used less frequently as specific hip shapes and conditions that could lead to abnormal forces and wear were defined [37]. It has long been recognized that acetabular dysplasia is associated with hip OA [38]. However, there are many variations in acetabular and femoral head shapes that may contribute to the development of OA [38]. Femoral acetabular impingement may be secondary to a pincer deformity wherein there is over coverage of the femoral head by the acetabulum or a cam deformity where there is extra bone on the anterolateral side of the distal head leading to a flattened cam-like appearance. The cam deformity is associated with earlier hip OA [39]. These cam hips often have decreased internal rotation due to impingement. There is a distinct correlation of the degree of femoral head deformity to the development of hip OA. The combination of deformity and loss of internal rotation is additive in their effect. On the other hand pincer deformity is not associated with the development of OA and may even be protective [40]. Significant variation in femoral head and acetabular version have been discovered to be associated with hip OA [41]. Radiographic statistical shape modelling has recently been employed to predict hip OA in females [42]. There are a variety of femoral head shapes that are recognized as a significant risk for hip OA [42]. It is interesting to note that there are racial and ethnic differences in a specific shape related to OA [43].

## 6 ASSOCIATED MUSCULOSKELETAL CHARACTERISTICS

It is obvious that associated skeletal disorders, such as osteoporosis, muscle weakness, muscle imbalance, and multiple osteoarthritic joints, and spinal disorders can influence treatment outcomes. A number of ways of defining these contributing factors have been developed and in some instances, the term phenotype is used.

It has been shown that systemic bone mineral density and bone mineral density in the vicinity of new insufficiency fractures are reduced [44]. This correlates with early OA wherein there is decreased periarticular bone density relative to systemic bone density [45]. The overall implication of most studies is that focal insufficiency fractures around the knee, formerly called spontaneous osteonecrosis of the knee are typically due to systemically insufficient bone. More recently the term subchondral insufficiency fracture of the knee has been applied to this disorder. However, a recent study has demonstrated in a US population that most patients with subchondral insufficiency fracture of the knee are not osteoporotic and, for their age, have above normal bone density [46]. The disconnection between low condylar bone density and a higher systemic bone density implies an important dynamic that may be related to early focal vascular signals.

In a large prospective multicenter study it was found that the combination of contralateral KOA, a baseline index KOA grade of 1, higher BMI, and a higher baseline Western Ontario and McMaster Universities Arthritis Index total scores were associated with greater likelihood to develop Kellgren and Lawrence grades of 3 or 4 within 5 years [47]. This parallels a Swedish study of an all Caucasian population that found that the relationship of bone mineral density, BMI, and lean body mass is different for hand OA versus lower limb joint OA [48]. Individuals with primary OA in the lower extremity have a phenotype with higher bone mineral density, higher BMI, and lower lean body mass content in contrast to no relationship in hand OA. The positive relationship was strongest for knees followed by hips and then ankles.

The degree of overall functional impairment will affect the treatment outcome for OA treatments. In Japan a functional assessment tool called “loco-check” includes the ability to put on a pair of socks while standing on 1 leg; stumbling or slipping at home; need to use a handrail when going upstairs; ability to cross a road before the traffic light changes; difficulty walking continuously for 15 minutes; finding it difficult to walk home carrying a shopping bag weighing about 2 kg; and difficulty doing housework requiring physical strength such as the use of a vacuum cleaner. This assessment will often reflect the combined effect of lumbar disorders, KOA and metabolic syndrome [49]. With functional limitations, the level of activity may be diminished to the point that it could have a negative impact on KOA [50].

A musculoskeletal comorbidity index has been developed to define the effect of multiple OA joints and lumbar spine disease on total knee joint replacement outcome. It has been found that after adjusting for age, sex, BMI, and Short Form-36 mental component summary score, moderate to severe preoperative pain in the contralateral knee (p = 0.013), ipsilateral (p = 0.014) and contralateral hip (p = 0.026), and low back (p < 0.001) was significantly associated with poorer function at 6 months after total knee replacement [51].

## 7 JOINT TISSUES CHARACTERISTICS

It has been highlighted that the paradigm of the focus on articular cartilage as the central feature and target for therapy in KOA has changed [52]. Other tissue processes may be more influential, including synovial inflammation and structural damage to the meniscus and subchondral bone. It is likely that there are a number of alternate pathways to OA [52, 53].

The advent of the MRI has made it possible to detect structural tissue changes that appear prior to the radiographic appearance of KOA. Research purposes now incorporate a system of whole organ involvement including bone, cartilage, meniscal, and synovial changes [54]. In an interpretation of the MRIs from the OA initiative, there were 355 patients who had an initial MRI with no radiographic KOA but who later developed radiographic KOA over 4 years of follow-up [9]. These patients were compared to case age- and sex-matched control individuals who did not develop radiographic OA. Cartilage lesions, bone marrow lesions, Hoffa’s fat lesions and combined effusion and synovium volume were scored on initial MRIs. This was contrasted to radiographic progression each year over the next 4 years. They found that the presence of specific structural features of MRI-detected joint damage 2 years prior to incident radiographic OA increased the risk of incident radiographic OA. One year prior to the radiographic change, bone marrow lesions and effusion-synovitis had the highest odds ratios. A similar study used data from the Foundation for the National Institutes of Health Osteoarthritis Biomarkers Consortium. The investigators found that for the 4-year period the number of cartilage surface regions affected, 6 or more, correlated highly with progression. Higher grade meniscal extrusion correlated to progression but not as great as articular cartilage change. The number of areas of bone marrow lesions correlated highly to progression. More advanced Hoffa fat changes correlated to progression more so than capsular distention. Changes in the degree of changes on MRI at 24 months also had specific correlations to subsequent radiographic KOA [55]. The combination of synovial and infrapatellar fat pad elaboration of degradative cytokines has been found to be important to the rate of articular cartilage degradation and pain [56]. Since pain is the target of most KOA therapies, the patient will set the greatest relevance in any pain modification in contrast to some estimated long-term joint preservation benefit.

## 8 DISCUSSION 

The use of phenotypes in KOA research can help us to understand variations in disease incidence, progression, and response to therapy. There have been specific recommendations by several research societies, but not all of them have been validated. In any given research project, investigators will need to use precaution in phenotype selection interpretations. Hence, investigators should be aware of the potential relevance of these phenotypes and make methodology decisions based on the available information.

The mixing of the term biomarker with MRI imaging and other imaging techniques can be confusing since the term biomarker connotes measurement of specific substances present in the tissues or circulation. Albeit the physical MRI categories are related to known biochemical changes, the use of the term biomarkers for MRI image categories is interesting in that some would consider these images to reflect phenotypes. Dr. Virginia Kraus noted in a personal communication the following:

I think of ‘progressor’ as the phenotype that is based on the current ‘gold’ standard biomarker of progression, *i.e.* the radiograph. By identifying baseline MR biomarkers that predict progression and/or change concurrently with progression based on radiograph (and in this case also pain progression) you are substituting one biomarker for another. Hopefully, the substitution is with a more sensitive biomarker (which we think is the case for many of these MR biomarkers). So I think of biomarkers as defining a phenotype and hopefully being able to distinguish and better define phenotypes with better biomarkers.

## CONCLUSION

Regardless of the terms used, the recording of different biochemical, imaging, and physical features will become increasingly important in OA research. There have been a number of key KOA phenotype clusters recommended by organizations in KOA research. The problem is the continuing need for validation of any given phenotype or phenotype cluster to the outcome. Despite this, investigators should be aware of the potential relevance of these phenotypes and make methodology decisions based on the available information.

## Figures and Tables

**Table 1:** Knee osteoarthritis phenotypes based on societal recommendations demographics, mechanics or injury, associate musculoskeletal conditions, joint components.

**Phenotype Group**	**References**	**PMIDs**
**Society Recommendations**	4, 11	25952342, 25596322
**Demographic characteristics**		
Height and weight	12-15,	26312543, 22662293, 18174218, 22595228
Sex	16	25447976
Race and ethnicity, knee	17-21	3381825, 8605262, 8317437, 17216685, 10555031
Age	22-28	1468204, 12209513, 3572588, 1954224, 11852229, 20129196, 10947951
**Mechanical characteristics**		
Knee mechanics	29-33	22995753, 26017348, 2010844, 27499317, 27185958
Knee injury	34-36	26521728, 21663852, 8998861
Acetabular and femoral head morphology	37-42	3780093, 27583059, 22730371, 23850552, 10608388, 26139655
Race and ethnicity, hip	42	26620089
**Associated musculoskeletal characteristics**		
Bone density and insufficiency fracture	44-48	14510760, 9818663, 25234658, 25280553
Functional limitations	49, 50	23114857, 26113741
OA other joints and spine	51	24132356
**Joint tissues characteristics**		
Knee tissue components	52-56	25940432, 20070426, 14872335, 27111771, 26455999
**Phenotype Validation**	1	28847624

## LIST OF ABBREVIATIONS

BMI: = Body Mass Index
KOA: = Knee Osteoarthritis
MRI: = Magnetic Resonance Imaging
OA: = Osteoarthritis
OARSI: = Osteoarthritis Research Society International
